# Heterogeneous expression of *DnaK* gene from *Alicyclobacillus acidoterrestris* improves the resistance of *Escherichia coli* against heat and acid stress

**DOI:** 10.1186/s13568-017-0337-x

**Published:** 2017-02-13

**Authors:** Xixi Xu, Lingxia Jiao, Xin Feng, Junjian Ran, Xinhong Liang, Ruixiang Zhao

**Affiliations:** School of Food Science, Henan Institute of Science and Technology, Hualan Road, Xinxiang City, 453003 Henan Province China

**Keywords:** Heterogeneous expression, Recombinant, Resistance, Heat and acid stress

## Abstract

*Alicyclobacillus acidoterrestris*, an acidophilic and thermophilic bacteria, is an important microbial resource for stress resistance genes screening. In this study, *DnaK* gene from *A. acidoterrestris* was subcloned to construct the recombinant plasmid pET28a–DnaK. The successful construction of the plasmid was verified by double-enzyme digestion and sequencing analysis. The recombinant plasmid was transformed into *Escherichia coli* BL21 and isopropy-β-d-thiogalactoside (IPTG) was used to induce recombinant *E. coli* to express *DnaK* gene. A 70 kD fusion protein was identified by SDS-PAGE, which suggested that *DnaK* gene from *A. acidoterrestris* was successfully expressed. The recombinant and wild BL21 were treated with high temperatures of 54, 56 and 58 °C at pH values of 5.0–7.0 to compare the effects of heterogeneous expression of the *DnaK* gene from *A. acidoterrestris* on the stress resistance. The experimental results showed that survival rate of recombinant BL21–DnaK has been improved considerably under heat and acid stresses in contrast with the wild BL21, and D-values of recombinant BL21 were 14.7–72% higher than that of wild BL21, which demonstrated that heterogeneous expression of *DnaK* gene from *A. acidoterrestris* could significantly enhance the resistance of host bacteria *E. coli* against heat and acid stresses.

## Introduction

During fermentation production process, microbial cell could encounter stresses from various aggressive conditions including high temperature, increased ethanol concentrations, high acidity and osmotic pressures, which could inhibit cell viability, lead to cell death, result in fermentation abnormal ceasing and thus reduce production efficiency etc. (Shafiei 2013; Bubulya 2011; Logothetis et al. 2007). Therefore, a major focus in food microbial research is to investigate ways to improve stress resistance of microbes cell in various severe conditions and maintain the high productivity of microbial in the industrial environment (Sugimoto et al. 2010; Andersson et al. 2010).

*Hsp70* possesses multiple biological functions and it has been well recognized as an induced chaperone protein by heat shock and other abiotic stresses, and *DnaK* can become over-expressed in cases of heat, high salt and alcohol to enhance tolerance of cell to harm and maintain normal metabolism (Groemping and Reinstein 2001; Seydlová et al. 2012; Di Pasqua et al. 2013). It has been shown that DnaK mRNA and DnaK protein of *Streptococcus mutants* were increased in response to acid shock (Jayaraman et al. 1997), and the strain’s capacity for growth in low-pH media becomes impaired, when DnaK levels of *S. mutans* are lowered via knockdown, strongly indicating the involvement of *DnaK* in acid tolerance (Lemos et al. 2007). Tomoyasu et al. (2012) found that *DnaK* knockout mutants of *Streptococcus intermedius* exhibited slow growth, thermosensitivity and accumulation of GroEL in the cell. The results imply that molecular chaperone most likely play a major role in the stress response of the bacteria.

*Alicyclobacillus acidoterrestris* is a genus of spoilage bacteria causing contamination in low pH foods such as apple juice and other beverage products due to its heat and acid resistance that enables survival from the traditional pasteurization procedures (90–95 °C for 30–60 s) (Smit et al. 2011). Due to the particular physiology, *A. acidoterrestris* has gained research interests focusing on detection and control in fruits juice (Mast et al. 2016; Cai et al. 2015; Bevilacqua et al. 2013), but gene regulation mechanism in the bacteria responding to high temperature and acidity are still unclear. It is reported that expression levels of *DnaK* gene in *A. acidoterrestris* were up-regulated rapidly under heat and acid stresses, and played significant roles for the survival of *A. acidoterrestris* in heat and acid conditions (Jiao et al. 2015). To validate the biological functions of *DnaK* gene *in A. acidoterrestris* resisting heat and acid stresses, recombinant *Escherichia coli* BL21 (DE3) expressing *DnaK* gene from *A. acidoterrestris* were constructed and the cell viability under heat and acid stresses were tested to evaluate effects of heterogeneous expression of the *DnaK* gene on stress resistance of recombinant *E. coli* BL21–DnaK, the objective of the study aims at the improvement of stress tolerance of microbes by transgenic technology in fermentation industry and provides theoretical basis for the development of the new microorganisms with high production and preferable stress tolerant properties.

## Materials and methods

### Bacterial strains and plasmids

The plasmid pET-28a(+) used for gene fusion construction was the pET System (Novagen, Germany). *E. coli* BL21 (DE3) strains were used as cloning hosts through this study. *Alicyclobacillus acidoterrestris* DSM 3922^T^ was purchased from Deutsche Sammlung von Mikroorganismen und Zellkulturen GmbH (German Collection of Microorganisms and Cell Cultures, Braunschweig, Germany).

### Construction of recombinant *E. coli* BL21 expressing *DnaK* gene

Amplification of *DnaK* gene (GenBank Accession No. HQ893543) was performed by PCR using genomic DNA of *A. acidoterrestris* as the template with the 5′ end primer (dnaKbF: 5′-GAGCTC*CCATGG*AGGAGGAACTTTCAGTGGCAAAAG-3′) containing an initiator ATG incorporated into an *Nco*I-site (shown in italics) and with the 3′ end primer (dnaKbR:5′-GTCGAC**CTCGAG**CTTCTGATCCTTGTCAACTTCGGTG-3′) to add an *Xho*I-site (in boldface type).

PCR amplification were conducted using the following protocol: 10 cycles of 94 °C for 5 min, 94 °C for 35 s, 61–55 °C for 30 s, 72 °C for 4 min, and 20 cycles of 94 °C for 30 s, 55 °C for 30 s, and 72 °C for 4 min and followed by 10 min at 72 °C. The PCR products were gel-purified and digested using *Nco*I/*Xho*I double digestion and subcloned into pET-28a(+) vector linearized by a double digestion with the same restriction enzymes to construct pET-28a(+)-DnaK recombinant plasmid, which was transformed into *E. coli* BL21. Bacterium liquid PCR and DNA sequencing were used to screen the positive recombinant clones.

### Induction and expression of DnaK protein

Recombinant *E. coli* BL21 were cultured in Luria–Bertani (LB) broth containing 50 mg/mL of kanamycin monosulfate at 37 °C overnight. Fifteen microliters of the overnight cultures were added to 15 mL fresh LB containing 50 mg/mL of kanamycin monosulfate and continued to incubate for 2–3 h and shaken at frequency of 250 rpm at 37 °C until mid-log phase (OD_600_ = 0.6). Then, half of the cultures is put into fridge for storage at 4 °C and used as control group (non-induced sample). The other half culture (approximately 7 mL) is added with isopropyl β-d-thiogalactopyranoside (IPTG) solution to a final concentration of 1 mM and induced at 37 °C for 3 h. 5 mL induced *E. coli* BL21 cultures (were centrifuged at 3000×*g*) for 10 min to harvest cells and supernatant is used as sample for LB broth. Then, the cells were resuspended in 50 mM phosphate buffer (pH 8.0, 500 mM NaCl, 1 mM EDTA) and lysed by sonication and the cell lysate was separated by centrifugation at 3000×*g* for 10 min into soluble protein extracts and sediment used as sample for SDS-PAGE analysis.

### Thermal inactivation of transformed *E. coli* at static temperatures

Cultures of *E. coli* BL21 were grown as described above. IPTG solution was added to mid-log phase cultures (OD_600_ = 0.6) to a final concentration of 1 mM, and incubation was continued at 37 °C for 3 h. After IPTG induction, the cells were centrifuged at 3000×*g* for 10 min to harvest the cells and then resuspended in fresh LB broth with different pH of 7.0, 6.0, 5.5 and 5.0, then immediately immersed in a water bath at 54, 56 and 58 °C respectively and treated for 10, 20, 30, 40 and 50 min.

The treated samples of 1 mL were taken every 10 min and decimal serial dilutions of the treated samples were prepared in a stroke-physiological saline solution and 100 μL of serial dilutions samples plated in triplicate onto LB plus kanamycin monosulfate plates. Plates were incubated for 24 h at 37 °C and colony forming units were enumerated to estimate cell viability (Velliou et al. 2011).

### Data analysis

Each sample collected in the experiment was analyzed in three replicates. Means of data were analyzed and compared by *T* test at P < 0.01 using statistical software (SAS version 9.4, USA). The experimental data (bacteria colonies number) were log-transformed and plotted as a function of time. Equation 1 was obtained by linear fitting and D-value was calculated.

1 $$N\left( t \right) = N(0) - \frac{t}{D}$$

with N (t) [Log (CFU/mL)] the stress resistant subpopulation, N (0) [Log (CFU/mL)] the initial cell population, t [min] the time and D [min] the time required to kill 90% of the bacteria.

## Results

### Construction of recombinant *E. coli* strain expressing *DnaK* gene

**Fig. 1:**
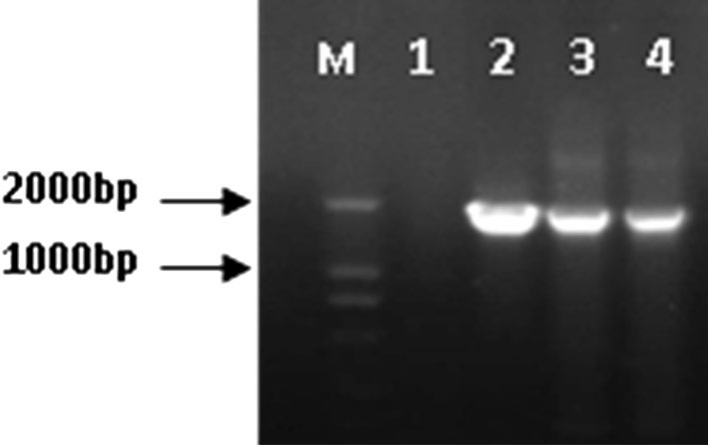
Single-colony PCR analysis of recombinant *E. coli* BL21-DnaK. *Lane M* DNA marker DL2000; *lane 1* negative control; *lanes 2–4* single-colony PCR products of recombinant *E. coli* BL21–DnaK

The coding region of *DnaK* gene was amplified with two primers designed and cloned into the pET-28a(+) plasmid to construct pET-28a(+)-DnaK expression vector for fusion protein expression. Then the recombinant plasmid pET-28a (+)-DnaK (Fig. 1) was transformed into *E. coli* and 3 clones were selected by growth in kanamycin monosulfate-containing medium.

**Fig. 2:**
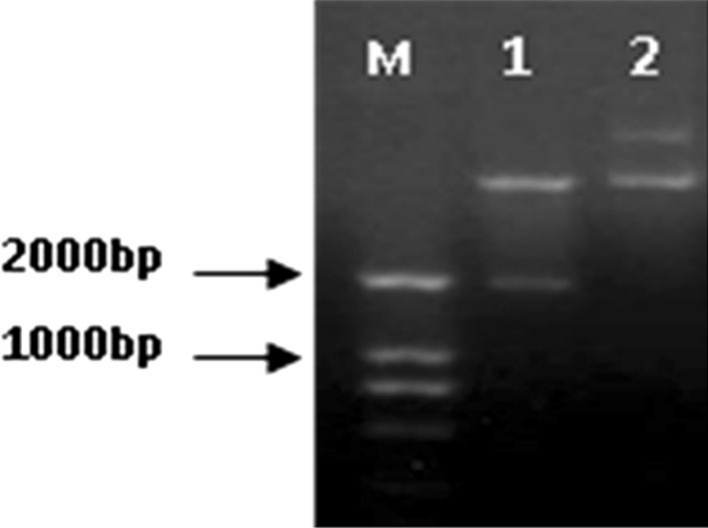
Double-enzyme digestion of recombinant plasmid. *Lane M* DNA marker DL2000; *lane 1* double-enzyme digestion products of recombinant plasmid; *lane 2* non-enzyme digestion of recombinant plasmid

The clones containing recombinant plasmid were identified by PCR, and then confirmed by double enzyme digestion with *Nco*I and *Xho*I and nucleotide sequencing. As shown in Fig. 1, the length of all PCR products was approximately 1854 bp and target fragment is inserted into vector with accurate enzyme digestion site (Fig. 2).

Based on nucleotide sequencing of the expression vectors produced, one clone (*E. coli* BL21–DnaK) was selected for expression of the recombinant protein. At the same time, the pET-28a(+) empty vector alone was also transformed into BL21 as the control group.

### Induction of fusion protein in recombinant *E. coli* cells

**Fig. 3:**
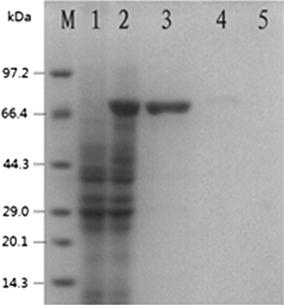
SDS–PAGE analysis of DnaK protein expression in recombinant *E. coli* BL21–DnaK. *Lane M* low molecular weight protein standard; *lane 1* whole-cell proteins of uninduced recombinant *E. coli* BL21–DnaK; *lane 2* whole-cell proteins of induced recombinant *E. coli* BL21–DnaK; *lane 3* supernatant of induced cells lysate; *lane 4* precipitation of induced cells lysate; *lane 5* LB medium

The supernatants of sonicated cells lysates from the 1 mM IPTG induced transformed cells were investigated to detect the presence of fusion protein by SDS–PAGE analysis. According to the construction (Fig. 1), the transformed *E. coli* cells should produce 70 kD fusion protein. A band about 70 kD fusion protein was clearly observed in gels containing cell supernatant, indicating this vector works normally and DnaK protein was expressed in the transformed *E. coli* BL21cells after the 3 h induction of IPTG (Fig. 3).

Moreover, content of soluble protein exceeds that of insoluble protein by a large margin and target protein is not detected in the supernatant of LB culture medium, which showed that DnaK fusion protein is mainly expressed as intracellular and soluble protein with no inclusion formation even accumulated at 37 °C.

### Stress resistance of recombinant *E. coli* cells expressing *DnaK* gene

**Fig. 4:**
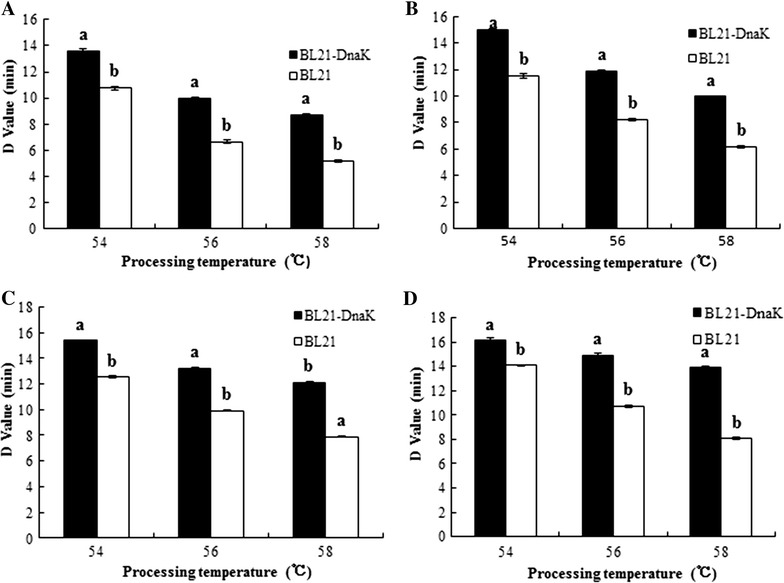
D-values of recombinant *E. coli* BL21–DnaK and *E. coli* BL21 at heat and acid stresses. **a** pH 5.0, **b** pH 5.5, **c** pH 6.0, **d** pH 7.0. The results represent the mean ± SD of three biological replicates. T-test is used to analyze the statistical differences*. Different capital letters* on the *top* of each column indicate the differences at the significant level of 0.01 between groups

In order to evaluate effects of induced expression of *DnaK* gene on stress resistance of host bacteria, recombinant *E. coli* BL21 expressing *DnaK* gene together with wild *E. coli* BL21 is treated with heat and acid stresses. Cell viability was measured by counting colony-forming units in triplicate LB plates and D-values were calculated using Eq. 1 to compare the heat resistance of recombinant *E. coli* BL21–DnaK with that of wild *E. coli* BL21 (Fig. 4).

As shown in Fig. 4, heat and acid stresses have significant effects on survive of wild and recombinant *E. coli* and result in declines of D-values, which implicated a quicker inactivation. The D-values for BL21–DnaK and BL21 in LB medium at different combination of temperature and pH-value ranged from 16.15 to 8.72 min and from 14.08 to 5.2 min, respectively. At the low pH of 5.0, D-values of *E. coli* BL21–DnaK were 25.69, 50.08 and 67.69% higher than that of wild *E. coli* BL21 under the heat stress of 54, 56, and 58 °C, respectively. Under the other treatment conditions of the same pH value and temperature, D-values of *E. coli* BL21–DnaK were higher than that of wild *E. coli* BL21 and ranged from 14.7 to 72%. The experiment suggested that *DnaK* gene expression not only led to a higher heat resistance of recombinant *E. coli* BL21–DnaK, but also significantly improved the resistance against acid stress.

## Discussion

For fermentation industry, the improvement of strain resistance against high temperature and acid could considerably reduce cooling requirements and prevent contaminative microbes, which avoided consumption of water and other resources and enhanced productivity, having important application significance and considerable commercial value (Gupta and Mukerji 2001). However, in adverse conditions of high temperature and strong acidity, a great many proteins in the microbe could be degenerated or aggregated, and finally led to the death of industrial microorganisms (Tissiéres et al. 1974). Hence, a key factor to improve the survival capacity and resistance of organism in stress conditions such as heat and acid is to prevent denaturation of intracellular proteins. Heat shock proteins could identify hydrophobic area on the surface of denatured protein, accelerate new protein folding and prevent irreversible agglutination reaction, thus effectively protect cell before damage (Horwitz 1992).

Extreme microorganisms in various habitats respond to external severe conditions by adapting their physiology through gene expression in order to survive and contain many unique functioning proteins itself. Research results showed that over expression of recombinant prefoldin deriving from *Pyrococcus horikoshii* OT3 could give *E. coli* tolerance of some organic solvents (Okochi et al. 2008), which suggested that heterogeneous expression of recombinant molecular chaperone gene from *Themophiles* played critical roles in enhancing the ability of host bacteria to resist the hostile circumstance. The heat shock protein genes have been successfully separated and researches have been conducted on inducing factors for the expressions and the relationship between over expression of heat shock proteins and heat resistance of organisms (Yeh et al. 1997).

Hsp70s, generally called chaperone protein DnaK in bacteria, were first discovered in fruit flies in the 1960s (Ritossa 1962) and responsible for essential functions in response to various biotic and abiotic stresses and involved in many cellular processes, including protein folding, protein translocation across membranes and regulation of protein degradation, which has been a hot research area in recent years (Yu et al. 2015). Studies in *Staphylococcus aureus* indicated that the DnaK system contributes to heat and oxidative stress tolerance, and DnaK protein refolding machinery plays an essential role in the stress responses (Singh et al. 2007). According to Simon et al. (1995), over expression of HSP70 could improve survival rates of murine fibroblasts exposed in ultraviolet rays and protect cells against DNA damage caused by ultraviolet C (Niu et al. 2006). Laksanalamai et al. (2006) found that molecular chaperone of extremely thermophilic bacteria *P. furiosus* had significant protective action to polymerase of Taq DNA under high temperature, which indicated that expression of chaperone extremophiles could improve tolerance of host cell against severe external environment.

Interest in the Hsp70 class of chaperones is growing because of the large variety of cellular processes in which they are involved, as well as their possible participation in environmental stress in vitro and pathological and physiological stresses in vivo (Gong and Golic 2006). For the objective of investigating the effects of heterogeneous expression of *DnaK* gene from *A. acidoterrestris* on stress resistance of *E. coli*, the research has established the expression carrier pET28a–DnaK, transformed into *E. coli* BL21, and then cell viability and resistance of recombinant and wild *E. coli* BL21 to high temperature and acidity was analysed and expressed in decimal reduction time (D-value). The research results showed that *DnaK* gene after induction of IPTG demonstrate a significant improvement of resistance of recombinant *E. coli* BL21–DnaK against high temperature and acidity, which mainly indicated by considerable increases in surviving rate and D-values of recombinant BL21–DnaK comparing to wild BL21. In microbiology, D-value refers to decimal reduction time and is the time required at a given temperature to kill 90% of the exposed microorganisms (Mazzola et al. 2003). Therefore, D-value of microorganisms is usually used in assessing microbial thermal resistance and thermal death time analysis (Berendsen et al. 2015). In our study, under heat stress at the same temperature, the D-values of microorganism decreased with declining pH-values. Moreover, under acid stress at the same pH-value, the D-values decreased with increasing temperature, which signifies that the higher the temperature, the faster the inactivation. The results of our study were in accordance with the general rules about effects of temperature and pH value on the thermal properties of microorganisms. However, the D-values of recombinant BL21–DnaK were 14.7 to 72% higher than that of wild BL21 under the same treatment temperature and acidity, which indicated *DnaK* gene expression in host bacteria *E. coli* BL21 could lead to a higher heat and acid resistance.

The *DnaK* gene is constitutive expression in *A. acidoterrestris* at the normal growth temperature of 45 °C, its expression is rapidly increased by 40% at a heat shock temperature of 70 °C for 5 min and induced 4.3-fold by acid stress in the low pH of 1.0 for 1 h (Jiao et al. 2015). The prominent expression of *Dnak* gene suggests that it may play an important role in thermotolerance and acid resistivity of *A. acidoterrestris*, and the results in the further study confirmed this supposition, which showed that heterogeneous expression of the *Dnak* gene could improve heat and acid resistance of recombinant *E. coli*. Changes in gene expression constitute the main component of the bacterial response to stress and environmental changes and molecular chaperones proteins are one of the main mechanisms (De Bruijn 2016). The function of *Dnak* gene from *A. acidoterrestris* improving by a large margin resistance of the host bacterium against acid and heat stresses might be related with special physiological potency of *A. acidoterrestris*, but the regulating mechanism needs further research to fully elucidate the roles of *Dnak* gene, the results of this study could contribute to the development of high endurance fermentation strains against adverse environments such as high temperature and acid in food industry.

## Abbreviations

*A. acidoterrestris*: *Alicyclobacillus acidoterrestris*
*E. coli*: *Escherichia coli*
IPTG: isopropy-β-d-Thiogalactoside
SDS-PAGE: SDS-polyacrylamide gelelectrophoresis
